# Cryptanalysis of a controller-independent controlled bidirectional QSDC protocol

**DOI:** 10.1371/journal.pone.0357035

**Published:** 2026-08-31

**Authors:** Chia-Wei Tsai, Yao-Chung Chang, Andy Su, I-Chun Chen, Ying-Hsun Lai

**Affiliations:** 1 Department of Computer Science and Information Engineering, National Taichung University of Science and Technology, Taichung, Taiwan; 2 Department of Computer Science and Information Engineering, National Taitung University, Taitung, Taiwan; 3 Department of Industrial and Business Management, Open University of Kaohsiung, Kaohsiung, Taiwan; China Medical University Taiwan, TAIWAN

## Abstract

A recent protocol by Cai et al. [*J. Korean Phys. Soc.* (2026)] proposed a controller-independent controlled bidirectional quantum secure direct communication (CICBQSDC) scheme based on four-particle cluster states, claiming provable unconditional security via Wyner’s wiretap channel theory. In this paper, we demonstrate that the protocol is vulnerable to a quantum Trojan-horse attack that is entirely consistent with the threat model assumed by the original authors; namely, the controller Charlie is an untrusted party with the same capabilities as the strongest possible eavesdropper. Specifically, this paper shows that Charlie uses Trojan-horse probes to steal $k_{\text{0}},\;k_{\text{1}},\;k_{\text{2}}$, and $k_{\text{3}}$ independently, and then he combines the measurement results and initial states of the four-particle cluster states to completely recover all four secret message bits encoded by Alice and Bob. Crucially, this attack introduces no disturbance to the legitimate qubits and therefore produces no increase in the ZX or XZ error rates, rendering it undetectable by the protocol’s security-check mechanism. This paper concludes that the unconditional security claim of Cai et al. fails to hold because it relies on a critical blind spot: a fundamental contradiction between modeling the controller as a maximally powerful eavesdropper and implicitly restricting their capabilities to a side-channel-free, ideal single-photon framework. This result highlights the general inadequacy of wiretap-channel-based security proof in the absence of rigorous physical-layer constraints, underscoring the critical need for implementation security in future QSDC designs.

## Introduction

Quantum cryptography harnesses the fundamental principles of quantum mechanics, including the no-cloning theorem, quantum indeterminacy, and quantum entanglement, to achieve information-theoretic security beyond the reach of classical methods. The field was launched in 1984 by Bennett and Brassard with the BB84 quantum key distribution (QKD) protocol [1], enabling two parties to share a secret key over an untrusted channel with provable security, and further enriched by Ekert’s entanglement-based E91 protocol [2] in 1991. Beyond QKD, the field has since expanded into quantum secret sharing [3], quantum key agreement [4], and quantum authentication [5]. Besides the aforementioned quantum communication protocols, a special protocol, Quantum Secure Direct Communication (QSDC) [6–8], has also been proposed, in which two participants can transmit confidential messages directly over the quantum channel without any pre-shared key. Foundational QSDC protocols include the Long & Liu scheme [6], the two-step EPR-based QSDC protocol of Deng et al. [7], and the single-particle DL04 protocol [8]. Building on these foundations, QSDC has evolved into unidirectional, bidirectional (quantum dialogue) [9], controlled [10], and most recently controller-independent controlled bidirectional variants [11,12], each offering different trade-offs among security, efficiency, and access control.

Recently, Cai et al. proposed a controller-independent controlled bidirectional QSDC (CICBQSDC) protocol based on four-particle cluster states, which not only requires controller authorization but also prevents the controller from learning the content of the exchanged messages [13]. In addition, Cai et al. provided rigorous security proof using Wyner’s wiretap channel theory and the quantum de Finetti theorem, claiming that their protocol is, to the best of their knowledge, the first cluster-state-based QSDC protocol achieving provable unconditional security. However, a critical blind spot exists in this security claim: there is a fundamental tension between the protocol’s assumed threat model and its reliance on an idealized physical framework. On one hand, the protocol explicitly designates the controller Charlie as an untrusted third party who is treated as the strongest possible eavesdropper Eve in the security analysis. On the other hand, the security proof is carried out entirely within the framework of ideal single-photon qubits subject to CPTP maps, without considering side-channel attacks that exploit physical degrees of freedom beyond the logical qubit Hilbert space, such as photon number, wavelength, or timing modes. This gap allows a class of attacks known as Quantum Trojan-horse attacks, in which an adversary injects additional photons into the legitimate participants’ encoding devices, to fall entirely outside the scope of the security proof while remaining fully consistent with the assumed capabilities of Charlie.

This study exploits the specific algebraic structure of the encoding unitary adopted in the CICBQSDC protocol to design a highly efficient quantum Trojan-horse attack. We show that by injecting ancilla qubits prepared in the state $\left|+\right\rangle =\frac{\text{1}}{\sqrt{\text{2}}}\left(|\text{0}\rangle +|\text{1}\rangle \right)$ into the quantum sequences transmitted to Alice and Bob, Charlie can directly extract each cover bit $k_{i}$ from the X-basis measurement results on the recovered ancilla qubits, with no information about the secret message bits $m_{i}\;$mixed into the result, where i∈{0,1,2,3}. Then, Charlie uses the measurement results of cluster states together with the recovered cover bits (i.e., $k_{i}$) to obtain the complete secret messages of Alice and Bob (i.e., $m_{i}$). The proposed attack introduces zero disturbance to the legitimate qubits, produces no increase in error rates, and thus is undetectable by the protocol’s existing security checks.

The remainder of this study is organized as follows. The Review of the CICBQSDC Protocol section reviews the relevant aspects of the CICBQSDC protocol and its security model. The Proposed Attack section presents the proposed Quantum Trojan-horse attack in detail. The Attack Analysis section explains why the attack evades all existing detection mechanisms. The Discussion section discusses the implications and suggests countermeasures. A conclusion of this study is given in the Conclusion section.

## Review of the CICBQSDC protocol

This section presents an overview of the CICBQSDC protocol proposed by Cai et al., followed by a summary of its encoding structure. Finally, we discuss the trust model associated with Charlie.

### Protocol overview

The CICBQSDC protocol involves three participants: Alice, Bob, and Charlie. Alice and Bob are the legitimate communicating participants, while Charlie acts as an untrusted controller. The CICBQSDC protocol proceeds in six steps as follows.

**Step 1.** Charlie prepares $N=\text{2}h_{\text{0}}+h_{\text{1}}+n$ four-particle cluster states, each randomly chosen from the 16 orthonormal cluster states $\left\{\left|C_{\text{0}\text{0}\text{0}\text{0}}\right\rangle ,\left|C_{\text{0}\text{0}\text{0}\text{1}}\right\rangle ,\ldots ,\left|C_{\text{1}\text{1}\text{1}\text{1}}\right\rangle \right\}$. He divides the particle sequence into two subsequences $P_{ac}$, consisting of the first and third particles (a and c) of a cluster state, and $P_{bd}$, consisting of the second and fourth particles (b and d).

**Step 2.** Charlie inserts decoy particles into $P_{ac}$ and $P_{bd}$ to form $P_{ac}+D_{ac}$ and $P_{bd}+D_{bd}$, where each decoy particle is randomly selected from {|0⟩,|1⟩,|+⟩,|−⟩}. Then, Charlie sends $P_{ac}+D_{ac}$ and $P_{bd}+D_{bd}$ to Alice and Bob via quantum channels, respectively.

**Step 3.** After confirming receipt, Charlie announces the positions and states of the decoy particles. Alice and Bob measure the decoys, compute the error rates, and abort if either exceeds a predefined threshold. They then perform additional security checks: they each select $h_{\text{0}}$ particle pairs, measure them in randomly chosen ZX or XZ bases, and then compare the results with Charlie’s announced initial states.

**Step 4.** Alice encodes her random check string $r_{A}$ and secret message $m_{A}$ onto $P_{ac}$ by applying the unitary operation given in Eq. (1).


$$\begin{array}{c} U_{k_{\text{0}}m_{\text{0}}k_{\text{1}}m_{\text{1}}}^{\left(ac\right)}=Y_{a}^{k_{\text{0}}}X_{a}^{m_{\text{0}}}⨂Y_{b}^{k_{\text{1}}}X_{b}^{m_{\text{1}}}, \end{array}$$ (1)

where $k_{\text{0}},\;k_{\text{1}}\in \left\{\text{0},\text{1}\right\}$ are Alice’s randomly chosen cover bits, and $\left(m_{\text{0}},\;m_{\text{1}}\right)$ is a 2-bit message packet. She sends the encoded sequence ${P^{\prime }}_{ac}$ back to Charlie.

**Step 5.** Bob similarly encodes $r_{B}$ and $m_{B}$ onto $P_{bd}$ using the following operation (i.e., Eq. (2)). Then, Bob also sends ${P^{\prime }}_{bd}$ back to Charlie.


$$\begin{array}{c} U_{k_{\text{2}}m_{\text{2}}k_{\text{3}}m_{\text{3}}}^{\left(bd\right)}=Y_{b}^{k_{\text{2}}}X_{b}^{m_{\text{2}}}⨂Y_{d}^{k_{\text{3}}}X_{d}^{m_{\text{3}}}, \end{array}$$ (2)

**Step 6**. Charlie performs cluster-state measurements on the corresponding pairs from ${P^{\prime }}_{ac}$ and ${P^{\prime }}_{bd}$, and publicly announces the measurement outcomes and the initial states of P. Alice and Bob then reveal their cover-bit positions and random check strings for integrity verification, apply error correction, and simultaneously decode each other’s secret messages.

### Encoding structure

When Alice and Bob apply their respective encoding operations to the initial cluster state $\left|C_{u_{\text{0}}u_{\text{1}}u_{\text{2}}u_{\text{3}}}\right\rangle$, the resulting state is $\left|C_{l_{\text{0}}l_{\text{1}}l_{\text{2}}l_{\text{3}}}\right\rangle$, where the indices satisfy:


$$\begin{array}{c} l_{\text{0}}=k_{\text{0}}⊕k_{\text{2}}⊕k_{\text{3}}⊕m_{\text{3}}, \end{array}$$ (3)

$$\begin{array}{c} l_{\text{1}}=m_{\text{0}}⊕m_{\text{2}}⊕k_{\text{3}}⊕m_{\text{3}}, \end{array}$$ (4)

$$\begin{array}{c} l_{\text{2}}=k_{\text{1}}⊕k_{\text{3}}⊕k_{\text{2}}⊕m_{\text{2}}, \end{array}$$ (5)

$$\begin{array}{c} l_{\text{3}}=m_{\text{1}}⊕m_{\text{3}}⊕k_{\text{2}}⊕m_{\text{2}}. \end{array}$$ (6)

After **Step 6**, the values $\left(l_{\text{0}},l_{\text{1}},l_{\text{2}},l_{\text{3}}\right)$ (Charlie’s measurement results) and $\left(u_{\text{0}},u_{\text{1}},u_{\text{2}},u_{\text{3}}\right)$ (the initial state) are all publicly known. However, the secret message bits $\left(m_{\text{0}},\;m_{\text{1}},m_{\text{2}},m_{\text{3}}\right)$ remain hidden because the cover bits $\left(k_{\text{0}},\;k_{\text{1}},k_{\text{2}},k_{\text{3}}\right)$ are unknown to Charlie, at least under the assumptions of the original security proof.

### Charlie’s trust model

Cai et al.’s paper [13] explicitly states that Charlie is an untrusted third-party controller, and that in the security analysis *the eavesdropper Eve can be the controller Charlie, with the same capabilities as Charlie*. The security proof then bounds the wiretap channel capacity $C_{W}=max\left\{I(AB:E)\right\}$ under the assumption that Charlie (as Eve) can apply an arbitrary unitary $U_{E}$ on the logical qubits and an auxiliary system. However, this model implicitly restricts Charlie’s attack to the single-photon qubit Hilbert space, excluding side-channel attacks that operate through additional physical modes such as photon number, wavelength, or timing.

## The proposed attack

In this section, we introduce the key algebraic observation which is used to extract Alice’s and Bob’s secret messages, and then the processes of the proposed attack are described. Finally, a formal statement is given to prove that Charlie can steal Alice’s and Bob’s secret messages by using the proposed attack.

### Key algebraic observation

The attack rests on the following elementary properties of the Pauli X and Y gates acting on X-basis eigenstates:


$$\begin{array}{c} \left|+\right\rangle =\left|+\right\rangle ,\;X\left|-\right\rangle =-\left|-\right\rangle , \end{array}$$ (7)

$$\begin{array}{c} Y\left|+\right\rangle =i\left|-\right\rangle ,\;Y\left|-\right\rangle =-i\left|+\right\rangle , \end{array}$$ (8)

where $X=(\begin{array}{cc} \text{0} & \text{1}\\ \text{1} & \text{0} \end{array})$, $Y=(\begin{array}{cc} \text{0} & -i\\ \text{1} & \text{0} \end{array})$, and $\left|\pm \right\rangle =\frac{\text{1}}{\sqrt{\text{2}}}\left(|\text{0}\rangle \pm |\text{1}\rangle \right)$. Eq. (7) shows that the X gate acts as the identity on the X-basis measurement result (producing only a global phase), while Eq. (8) shows that the Y gate flips the X-basis measurement result. Therefore, for an ancilla photon prepared in |+⟩, the combined action of $Y^{k_{i}}X^{m_{i}}$ is shown as follows:


$$\begin{array}{c} Y^{k_{i}}X^{m_{i}}\left|+\right\rangle =\left\{ \begin{array}{c} @l\left|+\right\rangle \;k_{i}=\text{0},m_{i}=\text{0}\;\\ \left|+\right\rangle \;k_{i}=\text{0},m_{i}=\text{1}\\ i\left|-\right\rangle \;k_{i}=\text{1},m_{i}=\text{0}\\ i\left|-\right\rangle \;k_{i}=\text{1},m_{i}=\text{1} \end{array}\right. \end{array}$$ (9)

An X-basis measurement on the ancilla thus returns |+⟩ if and only if $k_{i}=\text{0}$, and |−⟩ if and only if $k_{i}=\text{1}$, regardless of the value of $m_{i}$. Therefore, this study summarizes this as:


$$\begin{array}{c} \text{X}-\text{basis measurement result on ancilla}=k_{i}. \end{array}$$ (10)

### Attack process

- **Setup phase**: In Step 2, when Charlie sends the sequence $P_{ac}$ to Alice, he prepares, for each legitimate particle a (and similarly for a, b, and d), an additional ancilla photon $t_{a}$ in the state |+⟩, and injects it into the same or an adjacent physical mode (e.g., as an additional photon in the same pulse, at a slightly different wavelength or time window). He does the same for all four particles, yielding four ancilla photons $t_{a}$, $t_{c}$, $t_{b}$, and $t_{d}$ each prepared in |+⟩.
- **Encoding stage**: Alice’s physical encoding device (implemented by waveplates, phase modulators, or other linear-optical elements) applies the same unitary transformation $Y^{k_{\text{0}}}X^{m_{\text{0}}}$ to all optical modes passing through it, including the injected ancilla $t_{a}$. Consequently, after Alice’s encoding, the ancilla photon has evolved to:

$$\begin{array}{c} Y^{k_{\text{0}}}X^{m_{\text{0}}}{\left|+\right\rangle }_{t_{a}}, \end{array}$$ (11)

which by Eq. (9) is equal to |+⟩ if $k_{\text{0}}=\text{0}$ and i|−⟩ if $k_{\text{0}}=\text{1}$. The same applies to $t_{c}$, $t_{b}$, and $t_{d}$.

- **Recovery and measurement:** After Alice (or Bob) sends the encoded sequences back to Charlie, he uses spectral filtering, temporal gating, or photon-number-resolving techniques to separate and recover the four ancilla photons. He then measures each ancilla in the X-basis {|+⟩, |−⟩}, obtaining the following results:

$$\begin{array}{c} M_{t_{a}}=k_{\text{0}},\;M_{t_{c}}=k_{\text{1}},\;M_{t_{b}}=k_{\text{2}},\;M_{t_{d}}=k_{\text{3}} \end{array}$$ (12)

- **Secret message recovery:** Because Charlie now knows $k_{\text{0}},\;k_{\text{1}},k_{\text{2}},k_{\text{3}}$, he can substitute into Eqs. (3) to (6) to solve the secret message bits. Accounting for the initial state offset $u_{i}$, the solution is:

$$\begin{array}{c} m_{\text{3}}=l_{\text{0}}⊕u_{\text{0}}⊕k_{\text{0}}⊕k_{\text{2}}⊕k_{\text{3}}, \end{array}$$ (13)

$$\begin{array}{c} m_{\text{2}}=l_{\text{2}}⊕u_{\text{2}}⊕k_{\text{1}}⊕k_{\text{2}}⊕k_{\text{3}}, \end{array}$$ (14)

$$\begin{array}{c} m_{\text{0}}=l_{\text{1}}⊕u_{\text{1}}⊕k_{\text{3}}⊕m_{\text{2}}⊕m_{\text{3}}, \end{array}$$ (15)

$$\begin{array}{c} m_{\text{1}}=l_{\text{3}}⊕u_{\text{3}}⊕k_{\text{1}}⊕m_{\text{2}}⊕m_{\text{3}}, \end{array}$$ (16)

According to the above-mentioned calculations, Charlie obtains all four secret message bits per cluster state in a single measurement round.

### Formal statement

This section now formalizes the effect of the proposed Trojan-horse attack on the joint state of the system and clarify why it does not alter any of the error statistics used in the original protocol. As described in the Attack Process subsection, Charlie injects ancilla photons into optical modes that are distinct from those used to carry the legitimate four-particle cluster states. Let $H_{logical}$ denote the Hilbert space of the logical modes corresponding to the particles a, b, c, and d of each cluster state, and let $H_{ancilla}$ denote the Hilbert space of the Trojan-horse modes associated with the injected ancilla photons. We assume that these mode sets are orthogonal, so that the total Hilbert space factorizes as follows:


$$\begin{array}{c} H_{total}=H_{logical}⨂H_{ancilla} \end{array}$$ (17)

Before the attack, the state of the logical system in one round of the protocol can be described by a density matrix $\rho_{logical}$ on $H_{logical}$. When Charlie injects the ancilla photons in the state $\rho_{ancilla}$ and Alice and Bob subsequently apply their encoding operations $Y^{k_{i}}X^{m_{i}}$ uniformly to all optical modes, the overall state after encoding becomes


$$\begin{array}{c} \rho_{total}={\rho^{\prime }}_{logical}⨂{\rho^{\prime }}_{ancilla}, \end{array}$$ (18)

where ${\rho^{\prime }}_{logical}$ is the state of the logical modes, and ${\rho^{\prime }}_{ancilla}$ is the state of the Trojan-horse ancillas containing the information about the cover bits, as analyzed in the Attack Process subsection. Crucially, the encoding operations act as a product unitary of the form


$$\begin{array}{c} U_{total}=U_{logical}⨂U_{ancilla}, \end{array}$$ (19)

so that, in the absence of any further coupling between the logical and ancilla modes, the total state remains a tensor product.

The parameter-estimation measurements performed by Alice and Bob in **Step 3** of the CICBQSDC protocol, namely the ZX- and XZ-basis measurements on selected pairs of cluster-state particles, act only on the logical modes. Each POVM element corresponding to an outcome *x* can therefore be written as follows:


$$\begin{array}{c} M_{x}=M_{x}^{logical}⨂I_{ancilla}, \end{array}$$ (20)

where $M_{x}^{logical}$ is an operator on $H_{logical}$, and $I_{ancilla}$ is the identity of $H_{ancilla}$. As a result, all error statistics inferred from these measurements depend only on the reduced state of the logical system, which is obtained by tracing out the ancilla subsystem:


$$\begin{array}{c} {\rho^{\prime \prime }}_{logical}={Tr}_{ancilla}\left(\rho_{total}\right). \end{array}$$ (21)

This observation is summarized in the following lemma.

**Lemma 1**: Ancilla Orthogonality and Invariance of Error Statistics

Let $H_{total}=H_{logical}⨂H_{ancilla}$, and suppose that after a quantum Trojan-horse attack the joint state has the following product state:


$$\begin{array}{c} \rho_{total}=\rho_{logical}⨂\rho_{ancilla}, \end{array}$$ (22)

while all parameter-estimation measurements used by Alice and Bob have POVM elements of the form shown in Eq. (20). Then the reduced state of the logical system is unchanged,


$$\begin{array}{c} {\rho^{\prime }}_{logical}={Tr}_{ancilla}\left(\rho_{total}\right)=\rho_{logical}, \end{array}$$ (23)

and the outcome probabilities


$$\begin{array}{c} P_{x}=Tr\left(M_{x}\rho_{total}\right) \end{array}$$ (24)

are equal to those obtained from $\rho_{logical}$ alone. All error-rate statistics (including the ZX and XZ error rates) are completely unaffected by the presence of the Trojan-horse ancilla modes.

***Proof*:** We have


$$\begin{array}{c} {\rho^{\prime }}_{logical}={Tr}_{ancilla}\left(\rho_{logical}⨂\rho_{ancilla}\right)=\rho_{logical}Tr\left(\rho_{ancilla}\right)=\rho_{logical}, \end{array}$$ (25)

due to $Tr\left(\rho_{ancilla}\right)=\text{1}$. For any measurement result *x*,


$$\begin{array}{c} P_{x}=Tr\left(M_{x}\rho_{total}\right) \end{array}$$

$$\begin{array}{c} =Tr\left(\left(M_{x}^{logical}⨂I_{ancilla}\right)\left(\rho_{logical}⨂\rho_{ancilla}\right)\right) \end{array}$$

$$\begin{array}{c} =Tr\left(M_{x}^{logical}\rho_{logical}\right)Tr\left(\rho_{ancilla}\right) \end{array}$$

$$\begin{array}{c} =Tr\left(M_{x}^{logical}\rho_{logical}\right) \end{array}$$ (26)

Therefore, the presence of the ancilla modes does not change any of the probabilities observed by Alice and Bob in the parameter-estimation stage and therefore does not alter any of the error-rate statistics derived. From a physical perspective, this mathematical result is highly intuitive. Tracing out the ancilla subsystem reflects the physical reality that Alice and Bob’s standard detection equipment is effectively “blind” to the Trojan-horse photons, which occupy distinct physical modes such as different wavelengths or non-overlapping time bins. Furthermore, because the encoding operations apply independent unitary transformations to each mode (Eq. 19), they do not generate any quantum entanglement between the logical signal photons and the injected ancilla photons. In the absence of entanglement to correlate their states, ignoring the unmeasured ancilla modes, mathematically represented by the partial trace, leaves the reduced density matrix of the logical qubits completely undisturbed. Consequently, the physical measurements performed on the logical qubits yield the same error statistics as if the attack had never occurred.

Combined with the analysis in the Attack Process subsection, Lemma 1 implies that Charlie can extract the cover bits (i.e., $k_{\text{0}},\;k_{\text{1}},k_{\text{2}},k_{\text{3}}$) from the X-basis measurements on the Trojan-horse ancillas and subsequently recover the secret message bits (i.e., $m_{\text{0}},\;m_{\text{1}},m_{\text{2}},m_{\text{3}}$) all while leaving the ZX and XZ error rates observed by Alice and Bob unchanged. Consequently, the X-basis Trojan-horse attack is completely invisible to the original protocol’s security checks.

## Attack analysis

### Decoy-state check does not cover ancilla modes

In **Step 3**, the security check is performed only on particles at positions previously designated as decoy positions by Charlie. Alice and Bob measure only these labeled particles and compare results with Charlie’s announcements. The quantum Trojan ancilla photons are injected into the physical modes of the legitimate signal particles, not at decoy positions, and are therefore never subjected to the decoy-state check. Even if Alice or Bob were to measure a particle at a decoy position, the ancilla photon occupies a distinct mode (e.g., different wavelength or time bin) and would not be captured by a measurement targeting the logical qubit.

### Error rates remain unchanged

According to the Formal Statement subsection and Lemma 1, the legitimate qubit modes are entirely unaffected by the ancilla injection. The ZX and XZ error rates $\varepsilon_{ZX}$ and $\varepsilon_{XZ}$ which form the basis for the security parameter estimation in the original paper, are therefore identical to those in the absence of the attack. The secrecy capacity lower bound $C_{s}=C_{M}-C_{W}$ computed in the original paper remains formally positive, yet the actual mutual information between Charlie and the secret messages is $I\left(Charlie;\;m_{A},m_{B}\;\right)=\text{4}\;$bits per cluster state. The Wyner-based security analysis is thus rendered completely ineffective against this side-channel.

### Contradiction with the stated threat model

The original paper states that “*the eavesdropper Eve can be the controller Charlie and has the same capabilities as Charlie*.” Since Charlie is responsible for preparing and distributing all quantum states in **Steps 1** and **2**, operations that are entirely within his designated role, the injection of ancilla photons into the distributed sequences falls squarely within the capabilities of an untrusted Charlie. The attack therefore does not require Charlie to exceed his stated role; it only requires that his encoding devices not be constrained to single-photon inputs, an assumption that was never made explicit in the original protocol specification.

### External adversary with Trojan-horse capability

The proposed attack is not restricted to the controller Charlie alone. If an external adversary is able to inject and later recover Trojan-horse photons at the input and output ports of Alice’s and Bob’s encoding devices, then the same X-basis Trojan-horse strategy can also be applied by that adversary. In particular, by preparing ancilla photons in the state |+⟩, the adversary can extract the cover bits (i.e., $k_{\text{0}},\;k_{\text{1}},k_{\text{2}},k_{\text{3}}$) from X-basis measurements on the recovered ancillas, exactly as in Charlie’s attack. Because Charlie publicly announces the initial cluster-state labels and the measurement outcomes in Step 6, the external adversary can combine these publicly available classical data with the recovered cover bits to reconstruct the secret message bits $m_{\text{0}},\;m_{\text{1}},m_{\text{2}},m_{\text{3}}$. Therefore, the vulnerability exposed here should be understood more generally as a side-channel weakness of the protocol implementation, rather than as a flaw that can only be exploited by the controller. However, unlike Charlie, an external adversary must additionally possess physical access to the quantum channels near Alice’s and Bob’s devices to inject and retrieve the Trojan-horse ancillas.

### Impact of realistic imperfections

The cryptanalysis presented in the preceding sections assumes an ideal physical implementation where the Trojan-horse attack succeeds with certainty. However, in practical QSDC systems, physical imperfections will inevitably degrade the attack’s performance. Two primary factors are non-ideal detector efficiency and partial mode overlap:

- **Non-ideal Detector Efficiency**: In **Step 6** of the attack, Charlie relies on single-photon or photon-number-resolving (PNR) detectors to recover the states of the ancilla photons. Real-world detectors have a finite quantum efficiency η<1 and are subject to dark counts. If Charlie’s detectors fail to register the returning ancilla photon (due to channel loss or inefficiency), he cannot extract the corresponding cover bit $k_{i}$. Consequently, the attack degrades from a deterministic full-message recovery to a probabilistic partial-information extraction. Charlie’s success rate per 4-bit message packet scales with $\eta^{\text{4}}$, meaning the attack becomes significantly less effective over high-loss channels or with poor-quality detectors.
- **Partial Mode Overlap**: The theoretical model assumes the injected ancilla photons experience the exact same unitary transformations $\left(Y^{k_{i}}X^{m_{i}}\right)$ as the logical signal photons. In practice, if the ancilla is injected into a slightly different spatial mode or time bin, the unitary applied by Alice’s or Bob’s physical encoding devices (e.g., phase modulators) might not be perfectly uniform across all modes. This partial mode overlap can lead to an imperfect unitary evolution of the ancilla, reducing the visibility of Charlie’s X-basis measurement and increasing his bit error rate in extracting $k_{i}$. More critically, if the injection mechanism causes unintended interference or crosstalk between the ancilla and the logical modes within the encoding device, it could introduce random phase errors into the legitimate cluster states. Such disturbances would violate the assumption in **Lemma 1**, leading to a detectable increase in the ZX and XZ error rates monitored by Alice and Bob.

## Discussion

In this section, we demonstrate the failure of the controller-independent design goal in Cai et al’s protocol and propose practical countermeasures and solutions.

### Failure of the controller-independent design goal

One of the primary design goals of Cai et al.’s CICBQSDC protocol is that the controller Charlie cannot access the communication content [13]. Our attack demonstrates that this goal is not achieved in the presence of Trojan-horse side channels: Charlie can recover the complete 4-bit secret message per cluster state with certainty, without Alice or Bob being able to detect the intrusion.

### Countermeasures and solutions

To defend against this attack, the following hardware-level and protocol-level countermeasures should be considered.

- **Wavelength Quantum Filters** [14,15]: Install optical bandpass filters at Alice’s and Bob’s stations to reject photons outside the designated signal wavelength band, preventing the entry of ancilla photons at different wavelengths. This is highly effective at preventing the entry of ancilla photons injected at different wavelengths. The feasibility of deployment is extremely high, as these filters are standard, commercially available off-the-shelf components in modern optical communications. Moreover, the financial cost and integration overhead in practical QSDC systems are very low.
- **Photon-Number Monitoring** [16]: Use photon-number-resolving detectors or beam-splitter-based monitoring to detect multi-photon inputs before they reach the encoding device. This countermeasure is crucial for intercepting in-band Trojan-horse photons that bypass wavelength filters. While deploying true PNR detectors provides precise photon counting, it is currently technically demanding. Alternatively, a beam-splitter combined with standard single-photon avalanche diodes (SPADs) offers a highly feasible, albeit statistical, monitoring approach. True PNR detectors incur a high financial cost and require specialized cooling, whereas beam-splitter-based monitoring is moderately priced and much easier to integrate into existing systems.
- **Power Monitoring and Optical Isolators** [17,18]: Randomly activate power monitors or optical isolators at Alice’s and Bob’s input ports to detect and block unexpected optical power levels. Optical isolators are inherently effective at preventing the back-reflection of injected ancilla photons, while power monitors can detect abnormal energy spikes from bright Trojan pulses. Both are mature technologies with high deployment feasibility. These components are relatively inexpensive and are already considered standard security add-ons in practical quantum cryptographic implementations.
- **Revised Security Model**: Extend the security proof to include additional optical modes and photo number degrees of freedom, explicitly assuming that Alice and Bob are equipped with the above countermeasures. Under such assumptions, the adversary’s attack space can be formally restricted to single-photon qubit attacks, and the original Wyner-based proof would remain valid.

## Conclusion

This study has presented a quantum Trojan-horse attack on the CICBQSDC protocol of Cai et al. The attack exploits the fact that the X gate acts trivially on the X-basis eigenstate |+⟩ while the Y gate flips its X-basis measurement outcome, allowing Charlie to cleanly separate and extract the random cover bits $k_{i}$ from the X-basis measurement results on injected ancilla photons. Combined with the publicly announced cluster-state measurement outcomes, Charlie can completely recover all secret message bits per cluster state without introducing any detectable error. The attack is fully consistent with the untrusted-controller threat model assumed by the original authors, and it falls entirely outside the scope of their security proof.

The result of this study demonstrates that the unconditional security claimed by Cai et al. [13] holds only within the idealized model of single-photon qubits with no side channels and does not extend to practical implementations where Charlie controls the quantum source and distribution channel. We hope that this analysis will help clarify the security boundaries of the CICBQSDC protocol and motivate further work toward implementation-secure cluster-state QSDC, in which formal security proofs are conducted under realistic device assumptions inclusive of Trojan-horse side channels. Specifically, moving toward advanced theoretical frameworks, such as Measurement-Device-Independent (MDI) architectures or fully Device-Independent (DI) models, could provide a fundamental mitigation against such hardware-specific vulnerabilities, as they eliminate the need to trust the internal workings of the physical devices.
